# Computational Design of Nitrile Hydratase from *Pseudonocardia thermophila* JCM3095 for Improved Thermostability

**DOI:** 10.3390/molecules25204806

**Published:** 2020-10-19

**Authors:** Zhongyi Cheng, Yao Lan, Junling Guo, Dong Ma, Shijin Jiang, Qianpeng Lai, Zhemin Zhou, Lukasz Peplowski

**Affiliations:** 1 Key Laboratory of Industrial Biotechnology, Ministry of Education, School of Biotechnology, Jiangnan University, Wuxi 214122, China; zyCheng@jiangnan.edu.cn (Z.C.); 7170201011@stu.jiangnan.edu.cn (Y.L.); guojunling@jiangnan.edu.cn (J.G.); 7180201048@stu.jiangnan.edu.cn (D.M.); 6190205045@stu.jiangnan.edu.cn (S.J.); 6190205046@stu.jiangnan.edu.cn (Q.L.); 2 Jiangnan University (Rugao) Food Biotechnology Research Institute, Rugao 226500, China; 3 Institute of Physics, Faculty of Physics, Astronomy and Informatics, Nicolaus Copernicus University in Torun, Grudziadzka 5, 87-100 Torun, Poland

**Keywords:** nitrile hydratase, NHase, thermostability, catalytic activity, rational design, bioengineering, molecular dynamics

## Abstract

High thermostability and catalytic activity are key properties for nitrile hydratase (NHase, EC 4.2.1.84) as a well-industrialized catalyst. In this study, rational design was applied to tailor the thermostability of NHase from *Pseudonocardia thermophila* JCM3095 (*Pt*NHase) by combining FireProt server prediction and molecular dynamics (MD) simulation. Site-directed mutagenesis of non-catalytic residues provided by the rational design was subsequentially performed. The positive multiple-point mutant, namely, M10 (αI5P/αT18Y/αQ31L/αD92H/βA20P/βP38L/βF118W/βS130Y/βC189N/βC218V), was obtained and further analyzed. The Melting temperature (*T*_m_) of the M10 mutant showed an increase by 3.2 °C and a substantial increase in residual activity of the enzyme at elevated temperatures was also observed. Moreover, the M10 mutant also showed a 2.1-fold increase in catalytic activity compared with the wild-type *Pt*NHase. Molecular docking and MD simulations demonstrated better substrate affinity and improved thermostability for the mutant.

## 1. Introduction

Nitrile hydratase (NHase) is one of the most representative industrial enzymes [1,2]. Such a multimeric enzyme harboring either a low-spin non-heme Fe (III) ion or a non-corrin Co (III) ion at its active site is able to biotransform nitriles to their corresponding amides at room temperature and physiological pH [3]. To date, numerous attractions have arisen, both academic and commercial, due to the wide application of NHase in the industrial-scale production of acrylamide, nicotinamide, and 5-cyanovaleramide. Most recently, the potential of such a green catalyst in the asymmetric synthesis of valuable amide-building blocks of herbicides and medicines has also been exploited [4,5].

During the industrial biotransformation process, high thermostability and catalytic efficiency represent the hallmark properties of NHase. However, due to the exothermic nitrile hydration process and toxicity of organic substrates/products, most NHases exhibit poor stability. The NHases from all three generations of industrialized acrylamide production strains, *Rhodococcus* sp. N-774, *Pseudomonas chlororaphis* B23, and *Rhodococcus rhodochrous* J1, become unstable above room temperature, which results in an uncontrollable increase in energy cost to keep the hydration reaction temperature at a low level [6]. As a result, the industry still calls for robust NHases which can withstand harsh conditions and fulfil cost-effective strategies in industrial amide production.

To address these problems faced by NHase and meet the industrial requirements, protein engineering has emerged as an effective way. Directed evolution and rational design, two principal approaches of protein engineering, have been applied to modulate and design different enzymes, and rational design has been the preferred choice as it is less time-consuming and requires smaller mutant libraries [7]. Besides, with the rapid development of bioinformatics tools, the rational design of NHase has become more feasible and reliable. Till now, several rational/semi-rational design approaches have been utilized to improve the thermostability of NHase, including salt bridge introduction, thermostable fragment swapping, domain swapping, and subunit fusion. For instance, salt bridges were formed in NHase from *Rhodococcus ruber* TH by introducing charged amino acids such as Asp and Lys to its β subunit, resulting in enhanced thermostability. The modified NHase showed 50% of activity after 6 h, but at a relatively low temperature (37 °C) [8]. Moreover, the β-6th helix in NHase from *Aurantimonas manganoxydans* ATCC BAA-1229 was substituted in Pei’s studies by a thermophilic fragment of NHase from *Pseudonocardia thermophila* JCM3095 (*Pt*NHase). The modulated enzyme showed improved thermostability. The modified NHase exhibited 50% of activity after 100 min at 50 °C (under the same condition, wild-type (WT) showed 50% of activity after 60 min) [9]. However, exchanging protein fragments, as applied by Pei, is very challenging and not possible in all cases. Cui et al. used the protein fragment swapping method to increase thermostability [10]. Xia et al. constructed a thermostable prokaryotic NHase with only one polypeptide by fusing the α and β subunits of the NHase from *Pseudomonas putida* NRRL-18668 [11]. In both the abovementioned studies, improved enzymes showed only 40% of activity after 40 min at 50 °C [10,11]. These successful examples of well-tailored NHases have proved that understanding the structural basis and performing an in-depth analysis of structure-function correlation of NHase could help improve the thermostability and catalytic efficiency of such an enzyme. In this study, we showed that applying an online tool, that is easy to use for all proteins and which proposes introducing several point mutations, is helpful in designing enzymes with improved thermostability. Our newly designed *Pt*NHase showed even better thermostability improvement than those reported for natural NHase by Pei [9].

Co-type *Pt*NHase exhibits high thermal stability, whereas its catalytic activity is relatively low. However, compared with some thermophilic NHases reported so far, the catalytic activity of *Pt*NHase is acceptable. For example, NHase from *Bacillus* RAPc8 showed optimum activity at 60 °C; however, its activity is extremely low [12]. Immobilization of *Bacillus* RAPc8 cells could improve its catalytic performance and thermostability [13,14]. Besides, NHase from *Bacillus pallidus* Dac521 showed moderate thermostability up to 55 °C, whereas the activity is relatively low compared with that of *Pt*NHase [15]. As a result, tailoring *Pt*NHase to further improve its thermostability and catalytic efficiency could make it an ideal candidate for industrial production of amide products. In the present study, we modulated both thermostability and catalytic efficiency of *Pt*NHase using a rational protein engineering approach by employing site-directed mutagenesis of selective non-catalytic residues. We constructed a mutant containing ten mutated residues, and molecular dynamics (MD) simulations on the mutant and its wild-type (WT) *Pt*NHase were performed to grasp insights into the molecular basis of improved thermostability of *Pt*NHase. We showed results of thermostability of the mutational variant of *Pt*NHase designed and tested at first by bioinformatical and theoretical modeling tools. In the beginning, the new variant of *Pt*NHase was proposed by the FireProt server [16] and then through molecular dynamics simulations, improvement of thermostability was checked, and the influence of all mutations was explained.

## 2. Results and Discussion

### 2.1. Rational Design of a Potential Thermostable PtNHase Mutant

The crystal structure of WT *Pt*NHase has been solved and is available on the Protein Data Bank (PDB ID: 1IRE) [17]. The rational design was then performed to identify amino acid residues that would potentially improve the thermostability of WT *Pt*NHase. In the present study, the FireProt server [16] was selected as a rational design tool because such an online server could offer a reliable design of stable multiple-point mutants. As a result, the 1IRE crystal structure (dimer) was submitted to the FireProt online server to predict potential thermostable mutants through energy-based approaches. Totally, ten mutations (αI5P, αT18Y, αQ31L, αD92H, βA20P, βP38L, βF118W, βS130Y, βC189N, and βC218V) calculated by an energy-based approach (Figure S1) were exported.

**Figure 1:**
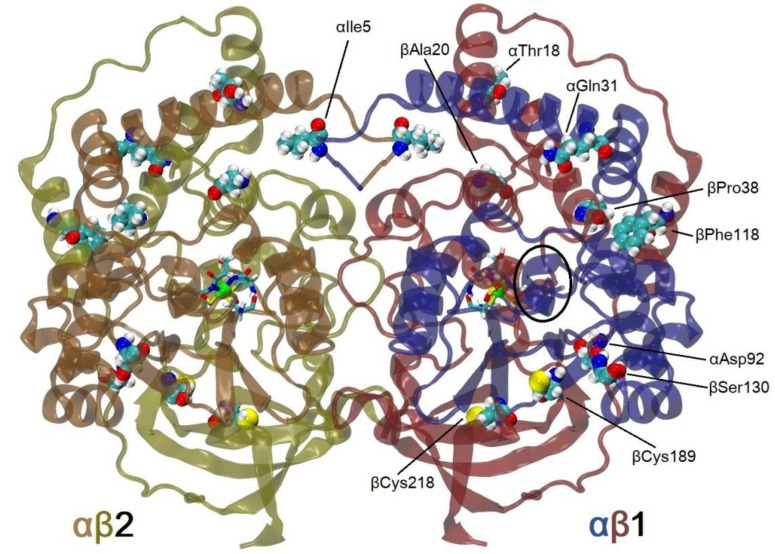
Structure of wild-type (WT) *Pseudonocardia thermophila* JCM3095 (PtNHase; PDB code: 1IRE) tetramer (biological assembly 1) with highlighted active site (licorice style), cobalt ion (green sphere), and 10 amino acids (vdW spheres) studied in this research. Blue and orange correspond to α subunit, red and yellow to β subunit. The αβ1 subunit present in the PDB file is shown in blue and red; subunit αβ2 from the biological assembly is shown in orange and yellow. In black ellipse, β178-184 α-helix is highlighted.

To be sure that the FireProt server predicted variants with better thermostability, before experiments, MD simulations were used to check the influence of the introduction of the 10 mutations to WT *Pt*NHase. The mutant thus constructed was named the M10 mutant, and it contained all the ten mutations predicted in one protein. In the case of NHase αβ dimers, partial denaturation of the enzyme was observed during long MD simulations (above 30 ns) (data unpublished). We decided to introduce for the first time 100 ns simulations of αββα tetramers—the smallest functional unit of such enzyme (Figure 1). Up to now, the longest MD simulations for NHase were at most 10 ns long [8,18], and in the case of Chen’s studies, apoenzyme without cobalt ion and post-translational modifications of cysteines were used.

**Figure 2:**
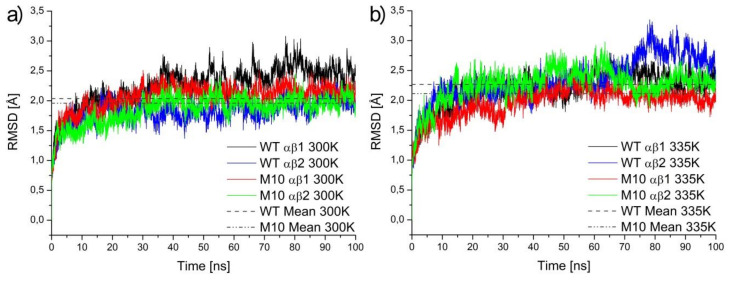
RMSD values for simulation of WT *Pt*NHase and its M10 mutant in 300 K (**a**) and 335 K (**b**).

An analysis of Root Mean Square Deviation (RMSD) plots of αβ subunits (Figure 2, αβ1 is symmetric to αβ2, although these subunits interact in the simulations, they can evolve quasi independently) showed, either in 300 K or 335 K simulations, that the M10 mutant was more stable than WT *Pt*NHase. The mean values of RMSD for the αβ subunits was 2.04 Å in the case of WT and 1.96 Å in the case of the M10 variant. After heating up to 335 K, RMSD values increased up to 2.27 Å and 2.12 Å for WT and M10 respectively, which meant that heating had a bigger influence on the structure of WT protein. One can see an increase in RMSD value after 70 ns for WT NHase. It was caused by the unfolding of the small surface helix in subunit β (residues from 178 up to 184, highlighted with an ellipse in Figure 1).

### 2.2. Construction of a Thermostable PtNHase Mutant

According to the information obtained from our rational design, the MD simulation results correlated well with the FireProt server prediction. As a result, the mutations listed above could be chosen as candidates for the design of the multiple-point mutations. Therefore, we constructed the corresponding M10 multiple-point mutant (αI5P/αT18Y/αQ31L/αD92H/βA20P/βP38L/βF118W/βS130Y/βC189N/βC218V) of *Pt*NHase (Figure S2). The catalytic activity of WT *Pt*NHase and its M10 mutant was measured by determining the amount of nicotinamide formed from 3-cyanopyridine under 25 °C. The specific activity of WT *Pt*NHase was 81.1 U·mg^−1^, whereas the activity of the M10 mutant reached 168.8 U·mg^−1^, showing approximately a 2.1-fold increase (Table 1). The Michaelis-Menten equation was used to characterize the kinetics of WT *Pt*NHase and its M10 mutant. Both enzymes were incubated with different concentrations of substrates. The *k*_cat_ value for the M10 mutant indicated a higher catalytic rate compared with the WT enzyme, which corresponded well with the activity data. The M10 mutant also showed a higher affinity for the substrate and thus, exhibited a higher catalytic efficiency compared with that of WT *Pt*NHase (Table 1).

### 2.3. M10 Mutant Shows Higher Thermostability

**Figure 3:**
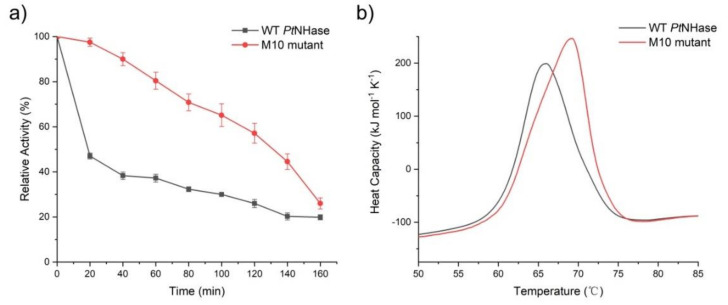
Half-life time determination and *T*_m_ value measurement of WT *Pt*NHase and its M10 mutant. (**a**) Half-life time determination of WT *Pt*NHase and its M10 mutant. (**b**) Nano DSC scan results of WT *Pt*NHase and its M10 mutant. The heat capacity results were analyzed using the Nano DSC analysis software, and the *T*_m_ values were calculated from a fitted curve.

To investigate the M10 mutant’s impact on the protein secondary structure, circular dichroism (CD) spectroscopy at the far-UV spectral region (190–250 nm) was measured. There were no substantial differences between the secondary structure of the WT and the M10 mutant, indicating that the ten mutations on *Pt*NHase had no destabilizing impact on the protein secondary structure (Figure S3). Subsequently, the half-life times of both WT *Pt*NHase and its M10 mutant were accessed. The enzymes were incubated at 65 °C and the activity of each enzyme was measured every 20 min. The M10 mutant retained above 50% of the initial activity after incubation for 2 h, whereas WT *Pt*NHase retained less than half of its initial activity after incubation for 20 min (Figure 3a). This was the best reported result so far for thermostability improvement of un-immobilized and natural NHase. Pei in his report enhanced the thermostability of NHase from *Aurantimonas manganoxydans* ATCC BAA-1229 from 60 min up to 100 min, measured at 50 °C (like in the present study) [9].

Furthermore, the thermodynamic stability of both WT *Pt*NHase and its M10 mutant was assessed using Nano differential scanning calorimeter (DSC). The thermal denaturation curves of the two enzymes are shown in Figure 3. The *T*_m_ values of WT *Pt*NHase and its M10 mutant were 66.0 °C and 69.2 °C (Figure 3b), respectively, indicating that the WT enzyme exhibited less thermostability, which was consistent with the thermostability levels observed in the half-life assays.

### 2.4. In Silico Docking Shows better Affinity of the Substrate with the M10 Mutant

**Figure 4:**
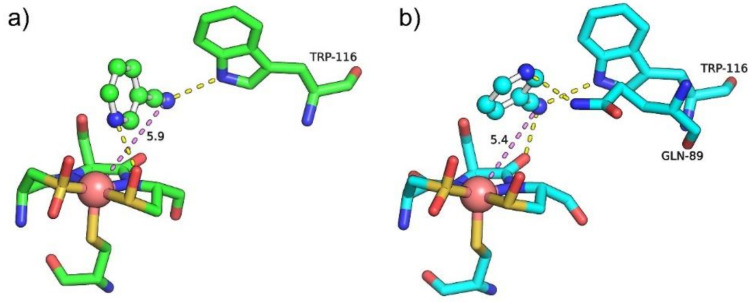
Molecular docking of 3-cyanopyridine into the active site of WT *Pt*NHase (**a**) and its M10 mutant (**b**). The active sites are presented as sticks. 3-Cyanopyridine is shown as balls and sticks. Yellow dash line represents the hydrogen bond formed between the ligand and the enzyme. Purple dash line represents the distance between the cobalt ion and the cyano nitrogen of 3-cyanopyridine.

Molecular docking was performed to predict the affinities of the ligand and the enzymes. AutoDock was applied to obtain the binding energies of 3-cyanopyridine toward both WT and M10 mutant. The M10 mutant showed lower binding energy (−4.98 kcal/mol) than WT *Pt*NHase (−4.18 kcal/mol). As the cyano group nitriles should coordinate with the metal ion at the active site before Cys114-SO^−^ attacks the coordinated nitrile to form a cyclic intermediate [19], the distance between the cobalt ion and the cyano nitrogen in the substrate is crucial for NHase catalytic activity. As shown in Figure 4, the distances between the cobalt ion and the cyano nitrogen of 3-cyanopyridine inside the M10 mutant was 5.4 Å (Figure 4b), whereas the distance between the cobalt ion and the cyano nitrogen of 3-cyanopyridine inside WT *Pt*NHase was longer (5.9 Å) (Figure 4a). These results were in good agreement with our previous docking studies [20]. The binding energy of 3-cyanopyridine to the M10 variant was only a little bit higher than that predicted in previous studies (−4.98 kcal/mol vs. −4.96 kcal/mol). However, the distance of CO-N was much lower in M10 (5.4 Å vs. 6.4 Å). It is worth noting that in our previous studies, we used an earlier version of AutoDock 3.0.5 [20]. 

### 2.5. MD Simulation Justified Higher Thermostability for M10 Mutant

**Figure 5:**
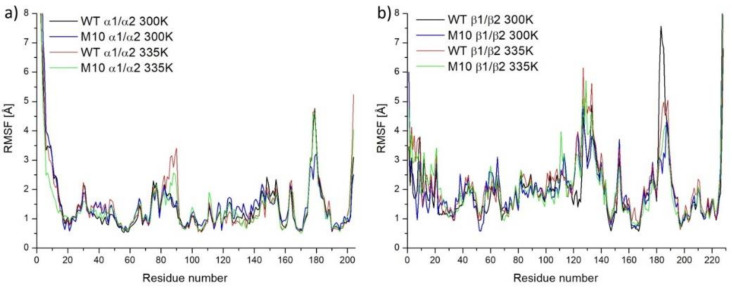
Averaged Cα atoms fluctuations for subunit α in 300 K and 335 K (**a**) and for subunit β 300 K and 335 K (**b**).

To gain better insight into the reasons that improved the thermostability of *Pt*NHase at the molecular level, we again carried out MD simulations on both WT *Pt*NHase and the M10 mutant. Root Mean Square Fluctuation (RMSF) showed which regions were more stable between WT *Pt*NHase and its M10 mutant. According to Figure 5, fluctuations of α subunit for both WT and the M10 mutant under 300 K were similar. After heating, two loop regions of the α subunit showed bigger fluctuations: residues 82–90 and 174–180 (Figure 5a). The first loop region in the M10 variant was more stable compared with that of WT *Pt*NHase. The second loop region of the α subunit which was exposed to a solvent showed stabilization after introducing mutations (in M10, 300 K).

In the case of the β subunit (Figure 5b), two regions showed low stability: residues 127–133 (long loop close to the active site cavity entrance) and amino acid residues 182–188 (also close to the active site cavity entrance). Interestingly, both regions stabilized each other by the salt bridge between αArg131 and βGlu188 (Figure S4). In the case of M10, these regions exhibited better stability.

Comparing the RMSF values of α and β subunits, it was obvious that the β subunit was less stable, which was in good correlation with other investigations [8,10,21]. Thus, the stabilization of the β subunit was crucial.

**Figure 6:**
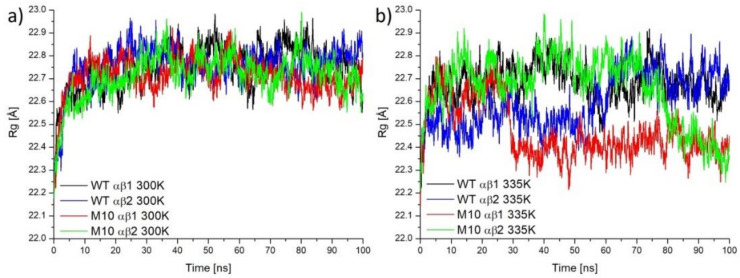
The radius of gyration for WT *Pt*NHase (black and blue lines) and M10 variant (red and green lines), calculated based on simulations in 300 K (**a**) and 335 K (**b**).

The radius of gyration (R_g_) represents the compactness of proteins. Proteins with lower R_g_ show better thermostability [22] and an analysis of R_g_ is very often used in the analysis of thermostability [23,24]. R_g_ values in the case of WT *Pt*NHase and its M10 variant showed similar values in the case of simulations in 300 K (Figure 6a). It was interesting that, in the case of simulations for the M10 variant in 335 K, both subunits αβ1 and αβ2 underwent conformational changes which resulted in bigger compactness, suggesting that the M10 variant was more stable in high temperatures (Figure 6b). The superposition of structures showing differences in R_g_ between two variants of NHase in two different time frames are shown in Figure S5.

**Figure 7:**
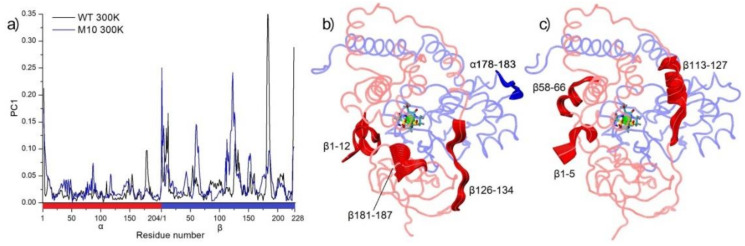
The first principal component (PC1) analysis of molecular dynamics (MD) simulations in 300 K. (**a**) Residue-wise loadings for PC1. Panels (**b**) and (**c**), movements of amino acids with loadings to PC1 bigger than 0.1 for WT and M10, respectively.

Principal component (PC) analysis gives insight into global movements and the contribution of all residues to these movements during the whole MD simulation. It is often used in the analysis of thermostability [23,25,26]. PC calculations based on MD simulations confirmed that the β subunit of *Pt*NHase was the most sensitive part to high temperature. The biggest loading in WT NHase in 300 K came from residues β181−190 (Figure 7). In the M10 variant, the influence of loadings of these amino acids was lower. The instability of this part and close-lying loop β126–134 was caused by breaking the αArg131–βGlu188 salt bridge (Figure S4) stabilizing region β126–134, which implied a bigger movement of whole α-helix β113–127. Interestingly, region β58–66 showed higher mobility in the M10 variant than that in WT NHase. In WT NHase, short part of chain α178–183 (loop region) showed higher mobility than the M10 variant.

**Figure 8:**
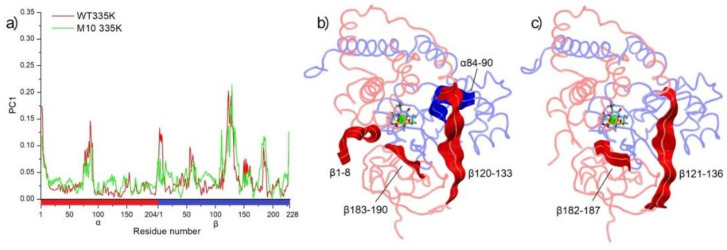
The first principal component (PC1) analysis of MD simulations in 335 K. (**a**) Residue-wise loadings for PC1. Panels (**b**) and (**c**), movements of amino acids with loadings to PC1 bigger than 0.1 for WT and M10, respectively.

After heating both systems, high movements of β182–190 could be observed in both variants of NHase, similarly like in region β120–136. At the high temperature, breaking of the αArg131–βGlu188 salt bridge could be noticed in both the WT and NHase variant (Figure 8). PC analysis showed that these regions of NHase were most critical in thermostability, suggesting focusing on these parts of enzymes in further research. In the case of WT NHase in 335 K, high mobility in the α chain could be observed for residues α84–90, where αGln89, a crucial amino acid for NHase activity, is located [27]. Bigger movements of this part of WT *Pt*NHase could be a reason for the lower catalytic activity of WT in higher temperatures.

Based on starting structures and structures captured every 10 ns (in total 11 structures for each case), constrained network analysis (CNA) was performed using the CNAnalysis Web Interface server [28,29], which can be used for thermostability estimating. The analysis showed that the rigidity order parameters were −0.58 ± 0.13 kcal/mol and −1.18 ± 0.35 kcal/mol, respectively, for WT NHase and M10 variant. This parameter can be converted to temperature. For WT, the temperature was determined to be 311.67 ± 2.67 K and for M10, the temperature was 323.64 ± 6.97 K [16], which indicated that the M10 variant should be more stable in high temperatures.

**Figure 9:**
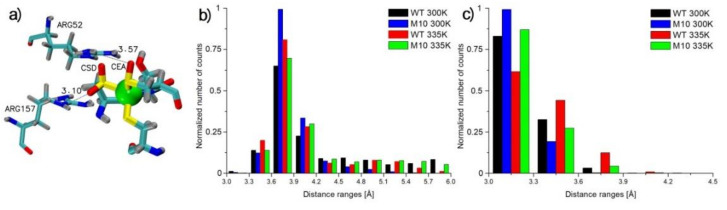
(**a**) Active site, βArg52 and βArg157 (based on crystal structure 1IRE). (**b**) Distance histogram between Cζ βArg52 and Oδ CEA113. (**c**) Distance histogram between Cζ βArg157 and Oδ1 CSD111.

Some of the most important amino acids in NHase are βArg52 and βArg157, coordinating the active site by hydrogen bonds with post-translationally modified cysteines (Figure 9a) [30]. After mutating one of these residues, NHase loses catalytic activity [31,32]. The distances between Cζ atoms of the two arginine residues and the modified active-site cysteines were analyzed. The crystallographic distance between Cζ βArg52 and Oδ CEA113 was 3.57 Å (Figure 9a). In MD simulations, this distance was determined to be between 3.6 Å and 3.9 Å. Such a distance was observed for the M10 variant frequently in 300 K. Very similar trends in distances were registered for WT in 300 K and M10 in 335 K, although in these cases, distances with high values (up to 6 Å) for WT in 335 K could also be observed (Figure 9b). The distance histograms correlated well with NHase activity at the low temperature, and the M10 variant had significantly higher activity than WT. The total activity of M10 at the high temperature after 160 min was at the level of WT at room temperature, and a similar behavior of distances for WT in 300 K and M10 in 335 K was observed. A similar correlation was observed for Cζ βArg157 and Oδ1 CSD113. The distance measured from the crystal structure was 3.10 Å (Figure 9a). An analysis of our MD simulations showed that the distance was between 3.0 Å and 3.3 Å in the case of M10 NHase in 300 K. After heating this variant up to 335K, distances were very similar to that observed for WT in 300 K. In the case of WT NHase in 335 K, higher distances were noticed compared with that of other simulations (Figure 9c). The observation of distances of both arginine residues with the active site showed that the structure of active site-Arg interactions were well kept in the case of M10 NHase in 300 K. After heating this version of the protein to 335 K, close vicinity of the active site and Arg residues behaved similarly to that of WT NHase in 300 K. The least stable situation was in the case of WT in 335 K. This observation correlated well with activities of the M10 variant at both low and high temperatures. 

### 2.6. Influence of a Particular Mutation on the Thermostability of PtNHase

#### 2.6.1. αI5P

**Figure 10:**
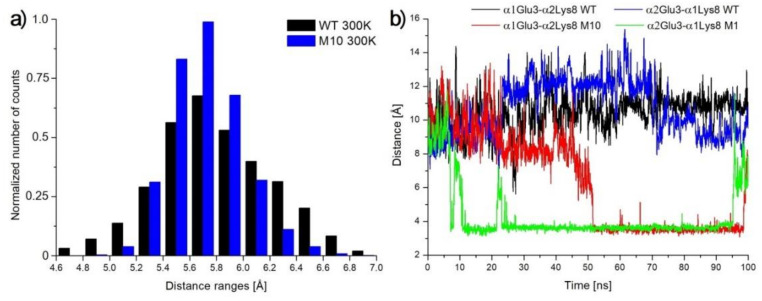
(**a**) Histogram of αAsn4 Cα and αArg7 Cα distances. (**b**) Plot of distances of αGlu Cδ–αLys Nζ in 300 K MD simulations.

The αIle5 in WT NHase is the only amino acid from the α1 chain that can interact with subunit αβ2 (and *vice versa*). It is located at the N-terminal loop of chain α. Substituting Ile to Pro causes part of the loop to become more rigid. In Figure 10a, distance histogram between αAsn4 Cα and αArg7 Cα is shown. In the case of the M10 variant, the most commonly observed distance was between 5.4 Å and 6 Å. In the case of WT, distribution was wider and distances more often were lower or higher than that of M10 NHase. This implied better orientation of α(1/2)Glu3, which could result in the creation of a salt bridge with α(2/1)Lys8 (Figure 10b). At temperature 335 K, such a salt bridge was rarely seen due to the thermal unfolding of α chain N-terminal part.

#### 2.6.2. αT18Y

In WT NHase, the side chain of αThr18 creates a stable hydrogen bond with the main chain’s carboxyl αGln14. αGln14 is located at the same α-helix as αThr18. This H-bond was observed in MD simulations in 300 K and 335 K (Figures S6a,b and S7). After mutation to bulky Tyr, the creation of such a hydrogen bond was not possible. After a few nanoseconds of simulation, OH group of αTyr18 created a stable hydrogen bond with βGlu28 (Figure S6c). This hydrogen bond between the two chains was present in both 300 K and 335 K simulations (Figure S7).

#### 2.6.3. αQ31L

**Figure 11:**
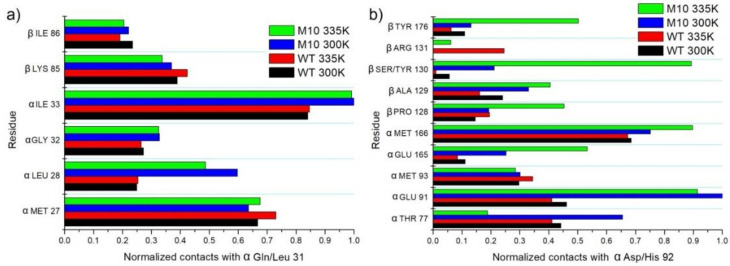
(**a**) Interactions of αGln/Leu31 with neighbor amino acids. (**b**) Interactions of αAsp/His92 with neighbor amino acids.

Substituting αGln into Leu in position 31 created a hydrophobic core with increased interactions with αLeu28 and αIle33 (Figure 11a). It is worth noting that interactions of Gln/Leu with βLys85 are typical hydrophobic interactions between C_β_H_2_ and C_γ_H_2_. In the WT enzyme, Gln does not create a H-bond with αLys31. NH_3_^+^ group from βLys85 is always oriented toward the solvent.

#### 2.6.4. αD92H

Mutating αAsp into His in position 92 changed the interaction network in close vicinity of such an amino acid. In the case of the M10 variant, a strong hydrophobic interaction was observed with αGlu91 (like in case Q31L); only CH_2_ groups from αGlu91 interacted in a hydrophobic way with His. Importantly, improved hydrophobic interactions of chain α with β were observed especially in the case of contacts of αHis92 with βTyr130 (also modified in M10) and with the hydrophobic fragment of βTyr176 (Figure 11b).

#### 2.6.5. βA20P

**Figure 12:**
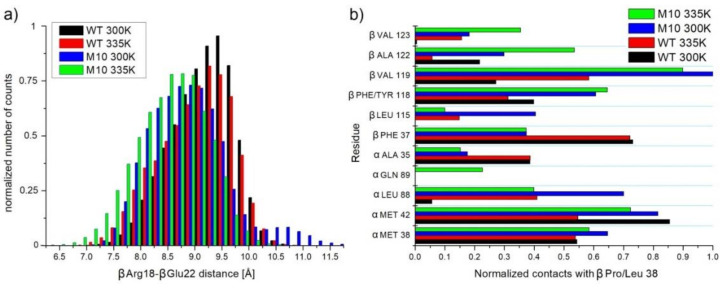
(**a**) Histogram of distances between Cα atoms of βArg18 and βGlu22. (**b**) Interactions of βPro/Leu38 with neighbor amino acids.

In WT NHase, βAla20 is located in the long N-terminal loop of the β chain. Residues from 1 to 13 of the β chain interact with other subunits. Residues 14–27 create a long loop exposed to solvent. Changing βLeu20 into Pro implied, in close vicinity to position number 20, that the loop was more curved and rigid. The most commonly observed distances between neighbor amino acids (Cα from βArg18 and αGlu22) in WT were between 8.75 Å and 9.75 Å. After mutation, this distance was shortened to 8–9 Å (Figure 12a). This substitution did not affect, however, the statistics of the salt bridges (βArg18–αGlu146, βArg18–αGlu199, and βGlu22–βArg192), which were very similar in all simulations. Neither the βAla20 side chain in WT nor the βPro20 side chain in M10 interacted with other amino acids. Both variants were exposed to a solvent. 

#### 2.6.6. βP38L

In many thermophilic proteins, increased content of prolines helps to stabilize loops. βPro38 is located in the middle of the helix (residues 27–43) [17]. Proline is known to destabilize helices or even break it. In the case of M10, this destabilizing amino acid was changed to a strongly hydrophobic Leu. Although interactions with βPhe37 and αAla35 were lost in part, a new hydrophobic core with βVal119, βPhe118, βAla122, βVal123, and αLeu88 was created (Figure 12b). Our previous studies of steered MD simulations showed an important role of βPhe37 in ligand gating [33]. RMSF calculated for all heavy atoms of βPhe37 (1.89 Å and 2.1 Å for WT and M10, respectively, in 300 K simulations) showed that this residue had more degrees of freedom in the case of the M10 variant. This may be one reason for the improved catalytic activity of M10 NHase.

#### 2.6.7. βF118W

**Figure 13:**
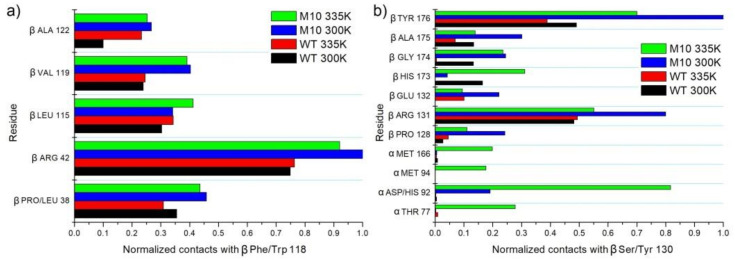
(**a**) Interactions of βPhe/Trp118 with neighbor amino acids. (**b**) Interactions of βSer/Tyr130 with neighbor amino acids.

The replacement of βPhe118 into Trp increased hydrophobic interactions with C_β_H_2_, C_γ_H_2_, and C_δ_H_2_ of βArg34, βVal119, and a little with βPro/Leu38 (Figure 13a).

#### 2.6.8. βS130Y

**Figure 14:**
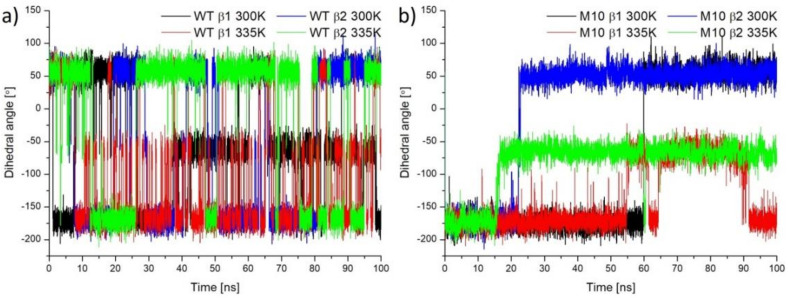
χ1 dihedral angle of βSer130 (**a**) in case of simulation for WT NHase and βTyr130 (**b**) in case of simulation for M10 NHase.

Position 130 is placed in a long loop (residues 127–142), which is close to the active site cavity entrance. Small hydrophilic βSer130 cannot create any hydrophobic interactions. Substituting it into a bulky Tyr with a hydrophobic aromatic ring changed the network of interactions. βTyr130 created hydrophobic interactions with βTyr179, βArg131, and in the case of simulations in 335 K additionally with αHis92, αThr77, αMet94, and αMet166 or βHis173 (Figure 13b), thanks to more degrees of freedom at a higher temperature. During MD simulations, frequent change of the χ1 dihedral angle of βSer130 could be observed (Figure 14a), although rotations of side group were quite often, the OH group did not create any hydrogen bond with the protein. In the case of βTyr130, we also could not observe any hydrogen bonds with the protein, but in this case, a bulkier side chain affirmed a more stable position and created hydrophobic interactions that were difficult to break even in the high temperature, which induced stable values of χ1 dihedral angle (Figure 14b).

#### 2.6.9. βC189N

βCys189 is placed close to short α-helix 178–183. In WT NHase, the side chain of βCys189 does not create any hydrogen bond. When βCys189 was substituted by Asn, a hydrogen bond was created with βThr181, which stabilized the short α-helix. In the case of WT NHase, unfolding of this α-helix could be observed in MD simulations in 300 K and 335 K. In the case of the M10 variant, unfolding could be observed only in one subunit in 335 K at the end of the simulation (Figure S8).

**Figure 15:**
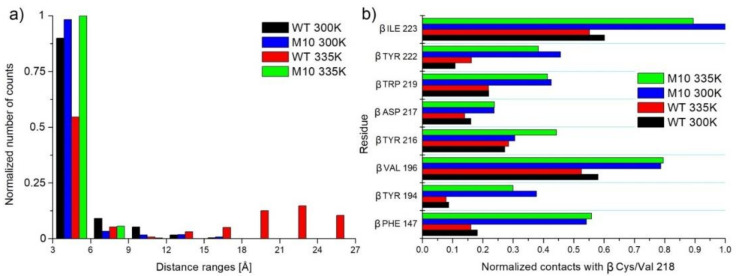
(**a**) Histogram of distances between Cζ atom of βArg131 and Cδ atom of βGlu188. Distance below 6 Å indicates the creation of the salt bridge between these residues. (**b**) Interactions of β Cys/Val218 with neighbor amino acids.

Moreover, α-helix stabilization together with better stability of the loop in case of the mutational variant in place 130 (S130Y) better oriented two close-lying amino acids, βArg131 and βGlu188, which resulted in more efficient salt bridge creation. In Figure 15a, histograms of distances between Cζ atom of βArg131 and Cδ atom of βGlu188 are plotted. Clearly, short distances below 6 Å, at which a salt bridge can be created, were more often in the M10 variant and the smallest number of distances below 6 Å was in the case of MD simulation of WT in 335 K. In the latter case, even long distances were observed (18–27 Å), showing the unfolding of one β chain at the high temperature. This observation was confirmed by PC analysis (Figure 6 and Figure 7).

#### 2.6.10. βC218V

In WT NHase, βCys218 is buried inside the protein between two beta-sheets. A modification to more hydrophobic Val resulted in the creation of a much bigger hydrophobic core. Increased interactions with βIle223, βVal196, βPhe147, βTrp219, βTyr222, and βTyr194 were observed (Figure 15b).

#### 2.6.11. M10 *Pt*NHase in the Context of Mechanisms of Increasing Thermostability

There are a few strategies achieved by evolution in increasing the thermostability of proteins. The most popular are: creating hydrophobic cores, introducing salt bridges, introducing disulfide bonds, making loops more rigid by introducing prolines, and increasing protein compactness [34,35,36,37]. FireProt is not able to design disulfide bonds; thus, such modifications were not used. 

Clearly, six mutations increased hydrophobic interactions: αQ31L, αD92H, βP38L, βF118W, βS130Y, and βC218V. The last two mutations listed above showed the biggest growth of hydrophobic cores. Mutations βS130Y and, to some extent, βP38L were able to create hydrophobic interactions between chains α and β. 

Mutations αI5P and βA20P prompted bigger rigidness of loops and as a result, in the case of the first variant, a new salt bridge (αGlu3–αLys8) can be created.

Mutations αT18Y and βC189N were involved in the creation of new hydrogen bonds, although it was not a common method used for increasing protein thermostability [37]. A new H-bond was created in the first case between amino acids from two different chains (αTyr18–βGlu28). In the case of the second mutation, a new hydrogen bond was created close in sequence, but it had a very big impact on preserving the structure of the small helix, most probably together with mutation βS130Y, where bigger stability of the χ1 angle was observed in the mutated NHase, and as a result the salt bridge βArg131–βGlu188 could be preserved. These mutations can improve the catalytic activity by keeping the proper structure of active site entrance and close-lying amino acids (arginine residues 52 and 157) from the β chain.

Besides, all mutations in M10 *Pt*NHase had a big influence on the compactness of the protein in higher temperatures (smaller values of R_g_ in case of the M10 variant in 335 K, Figure 6b), which results in better thermostability of the target enzyme.

## 3. Materials and Methods

### 3.1. In Silico Design of NHase

FireProt server version 1.0 was used [16,38].

As input for simulations, biological assembly 1 of 1IRE crystal structure was used [17]. Mutations to WT *Pt*NHase, designed by the FireProt server, were introduced using the psfgen mutate tool in NAMD 2.12 [39,40] package. Before the simulation, protonation states were determined using the PROPKA tool [41,42] implemented in the PDB2PQR server [43]. Next, structures were solvated with at least 10 Å in each direction and NaCl ions at a concentration of 0.15 mol/L with a neutralization option added. The starting simulation box had 93 Å × 111 Å × 82 Å and was composed of 78,220 and 78,271 atoms in the case of WT and M10 variant, respectively. For each variant, we performed 100 ns Langevin molecular dynamics simulation in 300 K and 335 K with 1 fs timestep, in atmospheric pressure, with long-range electrostatic interactions calculated using particle-mesh Evald summation. Before the main simulation, 1 ns water and ion equilibration, 1000 steps of energy minimization, and gradual heating up to a given temperature (60 ps in case of 300 K and 67 ps in case of 335 K) were performed. NAMD 2.12 [39,40] code with Charmm 27 [44,45] force field was used. Parameters for the nonstandard active site with cobalt and post-translationally oxidized cysteines were obtained based on extensive DFT/B3LYP/6-31G(d,p) and HF/6-31G* quantum calculations published in Peplowski’s PhD thesis, used for the first time in steered MD simulations for *Pt*NHase in 2008 [33]. The analysis was made using VMD 1.9.3 code [46] and homemade scripts. The principal component analysis was performed using the R Bio3D package [47,48]. Dockings of the substrate to the active site of NHase were performed using AutoDock 4.2 package [49,50]. The 3D structure of the 3-cyanopyridine ligand was obtained from the Cambridge Crystallographic Data Centre (CCDC) [51].

### 3.2. Protein Expression and Purification

Plasmid pET-24a (+) was used as a vector and *Escherichia coli* BL21 (DE3) was used for overexpression of NHase from *P. thermophila* JCM3095. The M10 mutant gene was synthesized by GENEWIZ Inc. (Suzhou, China) and cloned in plasmid pET-24a (+). The recombinant *E. coli* strain was cultivated in 2YT liquid medium containing kanamycin (50 mg/mL) under 37 °C. The expression of NHase was induced by adding IPTG to a final concentration of 0.4 mM and CoCl_2_·6H_2_O to a final concentration of 0.05 g/L, when the optical density at 600 nm (OD_600_) of cells reached 0.8 (cell mass was measured by using a UV-1800 spectrophotometer (MAPADA Instruments, Shanghai, China), and the wavelength was set to 600 nm). The cells were then continuously incubated at 24 °C for 16 h. Purification steps of the target enzymes were performed at 0–4 °C with 10 mM kalium phosphate buffer (KPB) buffer (8.02 mL of 1 M K_2_HPO_4_ buffer and 1.98 mL of 1 M KH_2_PO_4_ buffer in 1 L ddH_2_O) containing 0.5 mM dithiothreitol (pH 7.4). Cells were harvested (centrifugation at 6500× *g* for 15 min) and sonicated on ice. The supernatants were removed by centrifugation at 15,000× *g* for 15 min. Di-Ethyl-Amino-Ethyl (DEAE) Sephacel column (3 × 5 mL) (GE Healthcare UK Ltd., Buckinghamshire, UK) was first used and equilibrated with 10 mM KPB, and the protein was eluted with a linear gradient from 0 to 0.5 M KCl in KPB. The active fractions were then collected, concentrated to 1 mL by ultrafiltration, applied to a Superdex 200 10/300 GL column (GE Healthcare UK Ltd.), and equilibrated with 10 mM KPB, with a flow rate of 0.5 mL/min.

### 3.3. Enzymatic Assay

The specific activity of NHase was determined by the increase in the nicotinamide product. The reaction mixture contained 10 mM KPB (pH 7.4), 200 mM 3-cyanopyridine, and 10 μL of the appropriate amount of the enzyme solution (enzyme in 10 mM KPB buffer). The reaction was performed at 25 °C for 10 min. The concentration of amide was analyzed by HPLC equipped with a HITACHI C18 reverse phase column (solvent: acetonitrile/water = 1:2 (*v*/*v*)). The monitor wavelength was set to 215 nm. One unit (U) of NHase activity was defined as the amount of enzyme that produced 1 μmol nicotinamide per minute under the above assay conditions.

### 3.4. Measurement of the Kinetic Parameters of WT NHase and its M10 Mutant

The kinetic parameters for 3-cyanopyridine hydration by WT *Pt*NHase and its M10 mutant were determined in 10 mM KPB at 25 °C, and the concentration of enzymes was 0.2 mg/mL. The substrate concentrations were 10, 20, 50, 100, and 200 mM, and the reaction was terminated with 500 μL acetonitrile after 2 min.

### 3.5. Circular Dichroism (CD) and Thermal Denaturation Analysis

CD spectra of both WT *Pt*NHase and its M10 mutant were collected using MOS-450/AF-CD-STP-A (Bio-Logic, Grenoble, France) at a protein concentration of 0.2 mg/mL in 10 mM KPB. The ellipticity between 190 and 250 nm was measured, and the spectrum of a buffer blank was subtracted.

To measure the half-life of target enzymes, the purified NHase solution was pre-incubated in the absence of a substrate at 50 °C for different durations. The residual enzyme activity was then measured under standard conditions. The half-life (t_1/2_) of NHase was defined as the time when the residual activity retained 50% of its original activity at 0 min. 

The melting temperature (*T*_m_) was defined as the temperature when half of the protein was unfolded. Differential scanning calorimetry (Nano DSC, TA Instruments, New Castle, NY, USA) was used to capture changes in energy during the protein folding and unfolding processes. Enzymes were dialyzed against potassium phosphate buffer at a concentration of 0.3 mg/mL, and the instrument was scanned up and down from 25 to 100 °C using a 1 °C/min scan rate and scanned buffer alone to obtain background measurements. The heat capacity results were analyzed using the Nano DSC analysis software, and the *T*_m_ was calculated from a fitted curve.

## 4. Conclusions

In this article, we proved the rational design of proteins using bioinformatical and theoretical biophysics tools like servers dedicated to increasing protein thermostability and molecular dynamics simulations can be useful in predicting enzymes with improved thermal stability. Usually, blind attempts like error-prone PCR very rarely give the expected result. Many cases for checking in such an unwise method need large human and financial outlays. Rational design using cheap and fast methods like FireProt and molecular dynamics simulations (fast when access to high-performance computers (HPC) is available) can improve the design of proteins with requested properties, not only in the case of nitrile hydratase. Moreover, thanks to insight into detailed information about position evolution in time of all atoms in the biomolecules, which explained why the modified protein had better properties.

In our case, a new variant with 10 mutations of NHase was designed. This enzyme showed better thermal stability and, as a side effect, better activity. The M10 variant of *Pt*NHase can be easily used in the biotechnological process of green biotransformation of nitriles to amides.

## Figures and Tables

**Table 1:** Specific activity and kinetic parameters of WT *Pt*NHase and its M10 mutant.

Enzyme	*k*_cat_ (s^−1^)	*K*_m_ (mM)	*k*_cat_/*K*_m_ (s^−1^·mM^−1^)	Specific Activity (U·mg^−1^)
WT	107.9 ± 4.2	0.20 ± 0.03	539.5	81.1 ± 2.8
M10	165.1 ± 6.9	0.12 ± 0.02	1375.8	168.8 ± 5.3

## Supplementary Materials

The following are available online, Figure S1. Snapshot from FireProt server output with mutations energy information; Figure S2. SDS-PAGE of the purified WT *Pt*NHase and its M10 variant; Figure S3. Circular Dichroism (CD) spectroscopy at far-UV (190 to 250 nm) of the WT PtNHase and its M10 mutant; Figure S4. NHase (only αβ1 domain) with highlighted fragments with high fluctuations. αArg131-βGlu188 salt bridge is shown in black oval; Figure S5. Superposition of αβ1 structures showing changes in Rg. Figure S6. NHase with marked αThr18, αGln14 and βGlu28. αβ1 crystal structure panel (a); Figure S7. Distances between αThr18 Oγ atom and αGln14 carboxyl O atom (from main chain) (black and blue) in WT NHase; Figure S8. NHase with marked βCys189 in WT and βAsn189 in M10.

## Abbreviations

NHase	Nitrile hydratase
*Pt*NHase	NHase from *Pseudonocardia thermophila* JCM3095
M10	αI5P/αT18Y/αQ31L/αD92H/βA20P/βP38L/βF118W/βS130Y/βC189N/βC218V mutant of *Pt*NHase
WT	Wild-type
MD	Molecular dynamics
CD	Circular dichroism
RMSD	Root-mean-square deviation
RMSF	Root-mean-square fluctuation
PC	Principal component
CNA	Constrained network analysis
R_g_	Radius of gyration
CEA	Cysteine-sulfenic acid
CSD	Cysteine-sulfinic acid
H-bond	Hydrogen bond
KPB	Kalium phosphate buffer

## Notes

**Sample Availability:** Samples of the compounds are not available from the authors.
