# Supplementary material for: Computational Design of Nitrile Hydratase from Pseudonocardia thermophila JCM3095 for Improved Thermostability
Source: Molecules. 2020 Oct 19;25(20):4806. doi: 10.3390/molecules25204806 (PMC7587978; doi:10.3390/molecules25204806)
Supplement: Supplementary file 1 [file molecules-25-04806-s001.pdf]

Article

Computational Design of Nitrile Hydratase from *Pseudonocardia thermophila* JCM3095 for Improved Thermostability

Zhongyi Cheng [1], Yao Lan [1], Junling Guo [1], Dong Ma [1], Shijin Jiang [1], Qianpeng Lai [1], Zhemin Zhou [1,2,*] and Lukasz Peplowski [3,*]

[1] Key Laboratory of Industrial Biotechnology, Ministry of Education, School of Biotechnology, Jiangnan University, Wuxi 214122, China; zyCheng@jiangnan.edu.cn (Z.C.); 7170201011@stu.jiangnan.edu.cn (Y.L.); guojunling@jiangnan.edu.cn (J.G.); 7180201048@stu.jiangnan.edu.cn (D.M.); 6190205045@stu.jiangnan.edu.cn (S.J.); 6190205046@stu.jiangnan.edu.cn (Q.L.)

[2] Jiangnan University (Rugao) Food Biotechnology Research Institute, Rugao 226500, Jiangsu, China

[3] Institute of Physics, Faculty of Physics, Astronomy and Informatics, Nicolaus Copernicus University in Torun, Grudziadzka 5, 87-100 Torun, Poland

* Correspondence: zhmzhou@jiangnan.edu.cn (Z.Z.); drpepe@fizyka.umk.pl (L.P.)

Energy mutant: $-30.41 kcal/mol$ (10mutations)			
**Chain**	**Mutation**	**FoldX**	**Rosetta**
A	I5P	-1.34	-2.40
A	T18Y	-1.51	-4.57
A	Q31L	-1.43	-2.90
A	D92H	-1.98	-2.17
B	A20P	-1.08	-2.83
B	P38L	-1.24	-2.82
B	F118W	-1.33	-2.50
B	S130Y	-1.05	-2.38
B	C189N	-1.79	-3.04
B	C218V	-2.31	-2.91

Figure S1. Snapshot from FireProt server output with mutations energy information (M10 variant).

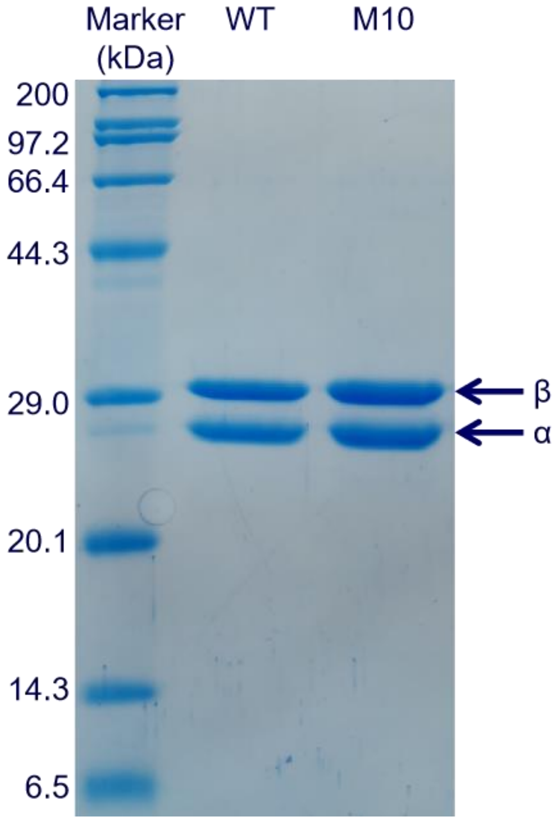

Figure S2. SDS-PAGE of the purified WT *Pt*NHase and its M10 variant.

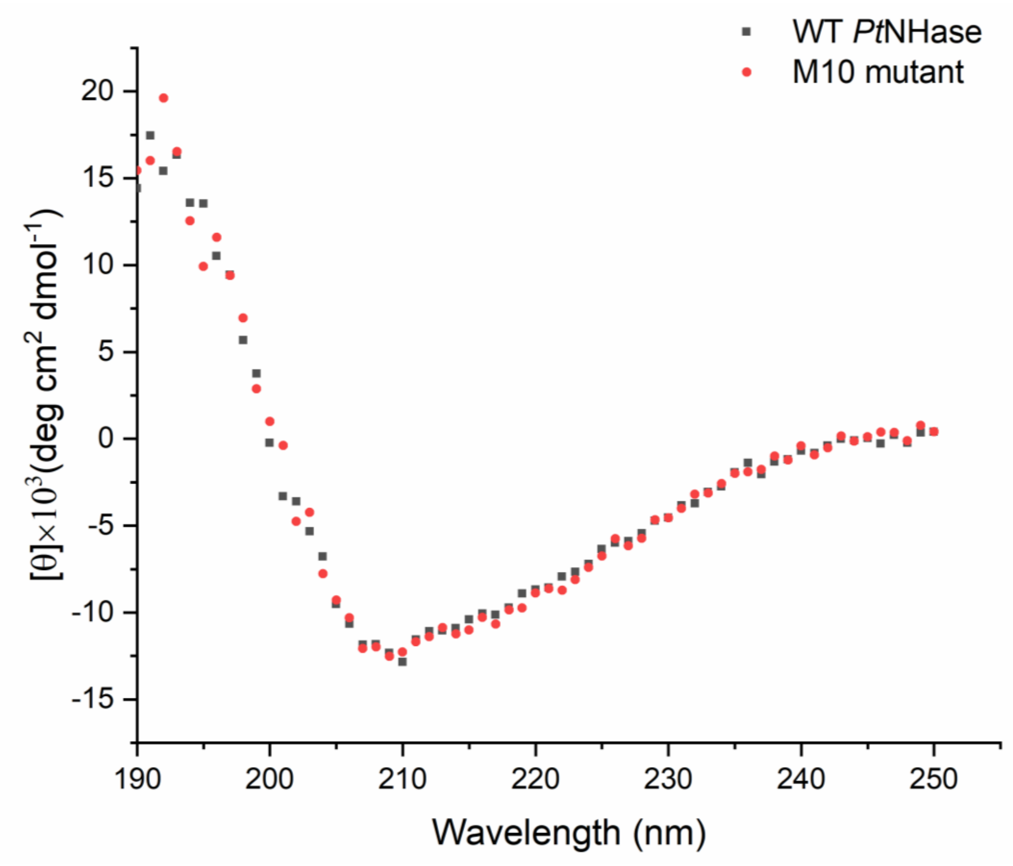

Figure S3. Circular Dichroism (CD) spectroscopy at far-UV (190 to 250 nm) of the WT PtNHase and its M10 mutant

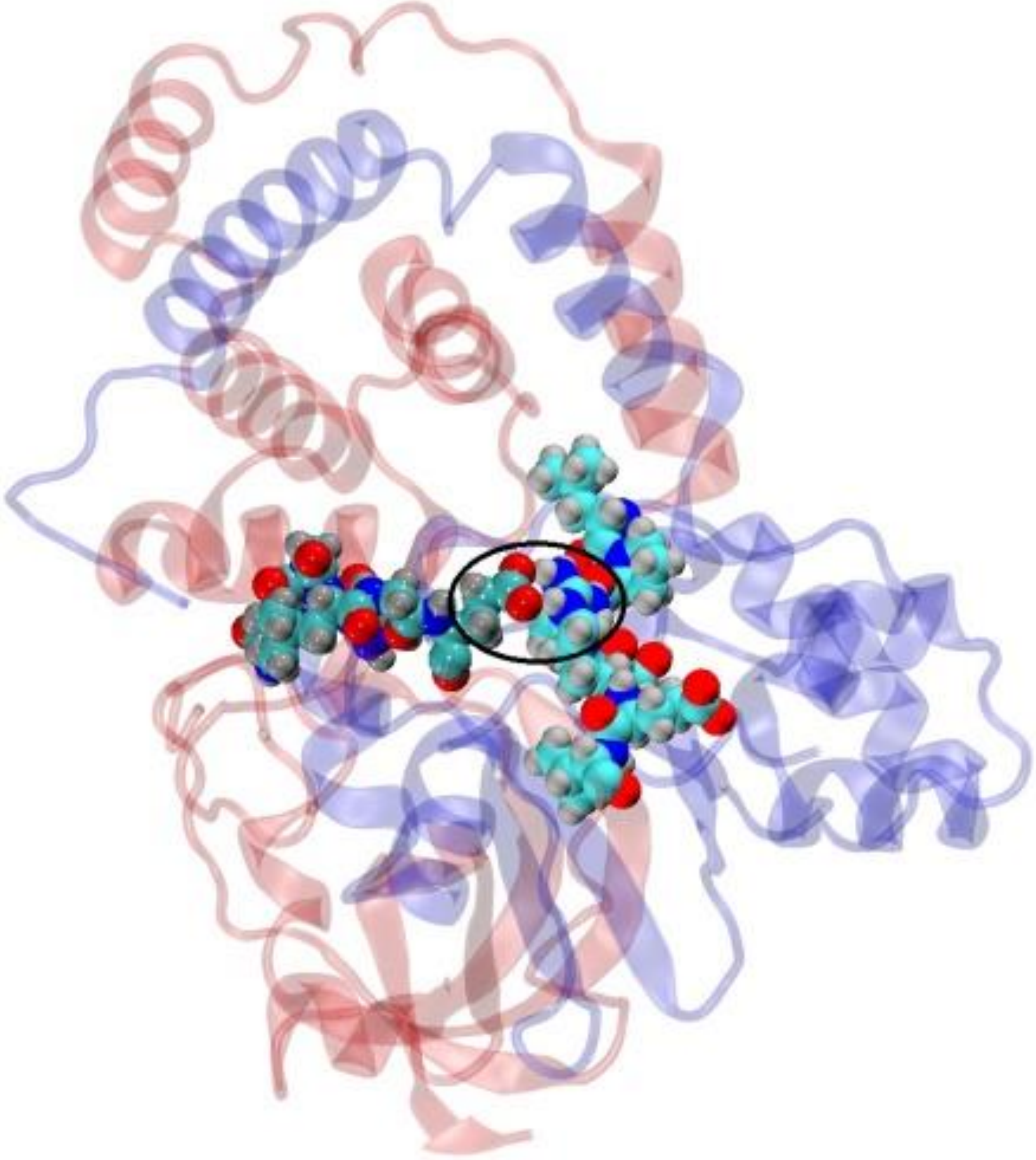

Figure S4. NHase (only $\alpha\beta1$ domain) with highlighted fragments with high fluctuations. αArg131-βGlu188 salt bridge is shown in black oval.

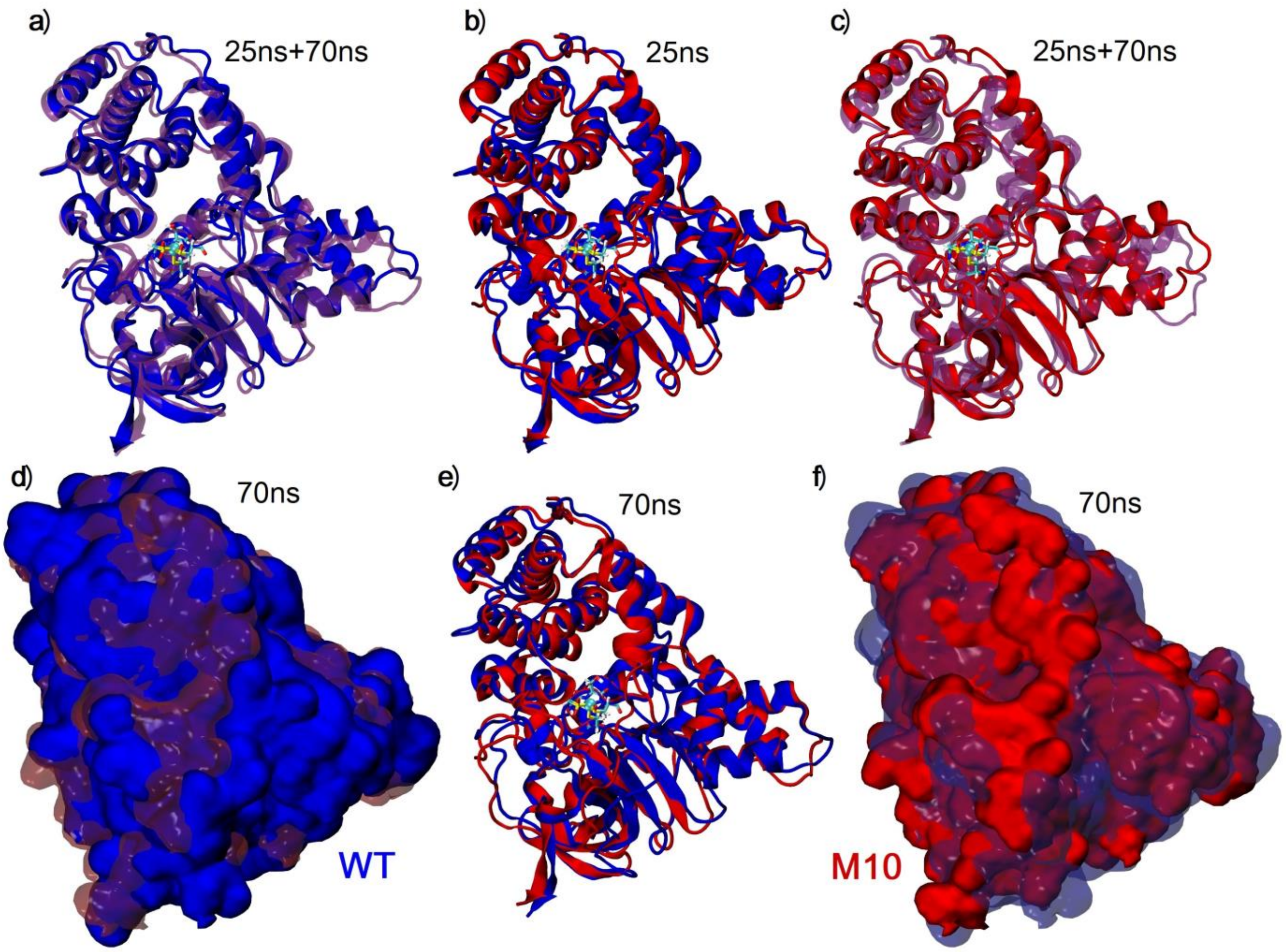

Figure S5. Superposition of αβ1 structures showing changes in Rg. WT (blue) and M10 (red) NHase structures after 25ns (panel b)) and after 70 ns (panel e)) are presented. In the panel b) structures are very similar (high Rg value). In the panel e) bigger compactness of backbone for M10 NHase can be observed. In panels a) and c) structures after 25 ns are shown respectively for WT (blue) and M10 (red). In the transparent magenta color (panels a) and c)) changes of structure after 70 ns can be observed. Panels d) and f) show superposition of surfaces of WT NHase (blue) and M10 (red) after 70 ns. In the panel d) in transparent red color M10 NHase is shown. In the panel f) in transparent blue color WT NHase is shown. One can observe surface of WT NHase, and thus Rg is bigger than in case of M10 variant.

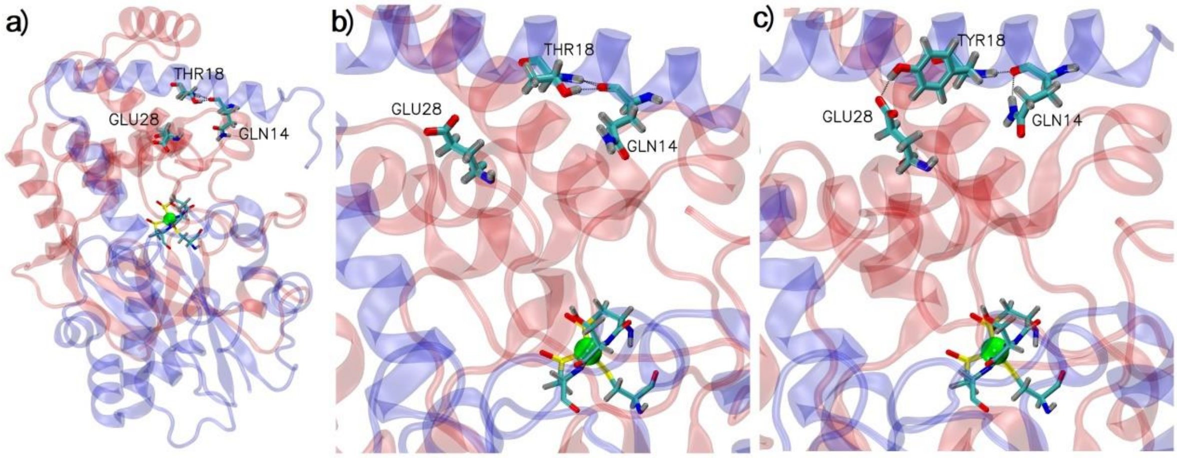

Figure S6. NHase with marked αThr18, αGln14 and βGlu28. $\alpha\beta1$ crystal structure panel (a). In blue α and β chain are shown in blue and red respectively. In the panel (b) H-Bonds between αThr18 and αGln14 Main chain after 100ns MD simulation is shown. In the panel (c) H-Bonds between αTyr18 and βGlu28 after 100ns MD simulation is shown.

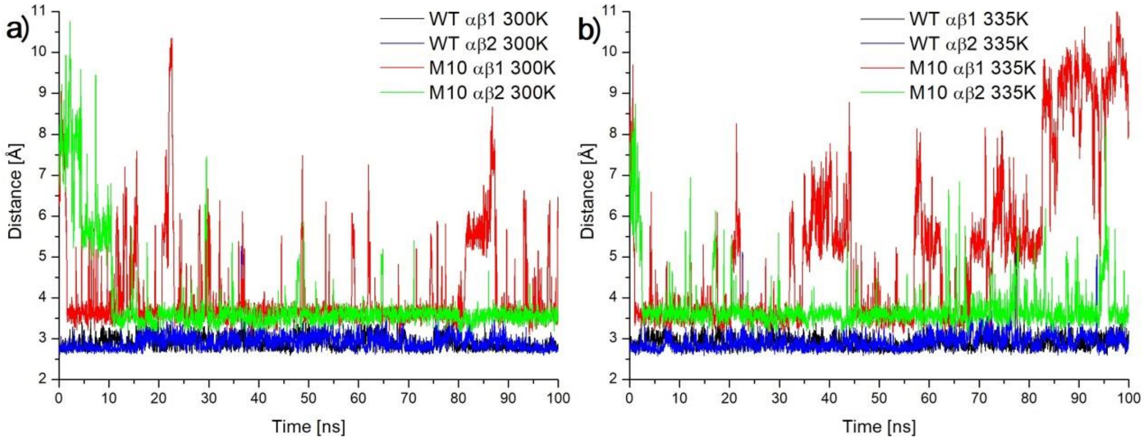

Figure S7. Distances between αThr18 Oγ atom and αGln14 carboxyl O atom (from main chain) (black and blue) in WT NHase. Distance below 3.5 Å indicates hydrogen bond. In red and green distance between αTyr18 Oh atom and βGlu28 Cδ atom. Distance below 4 Å indicates hydrogen bond. Panel (a) results from simulations in 300K, panel (b) results from simulations in 335K.

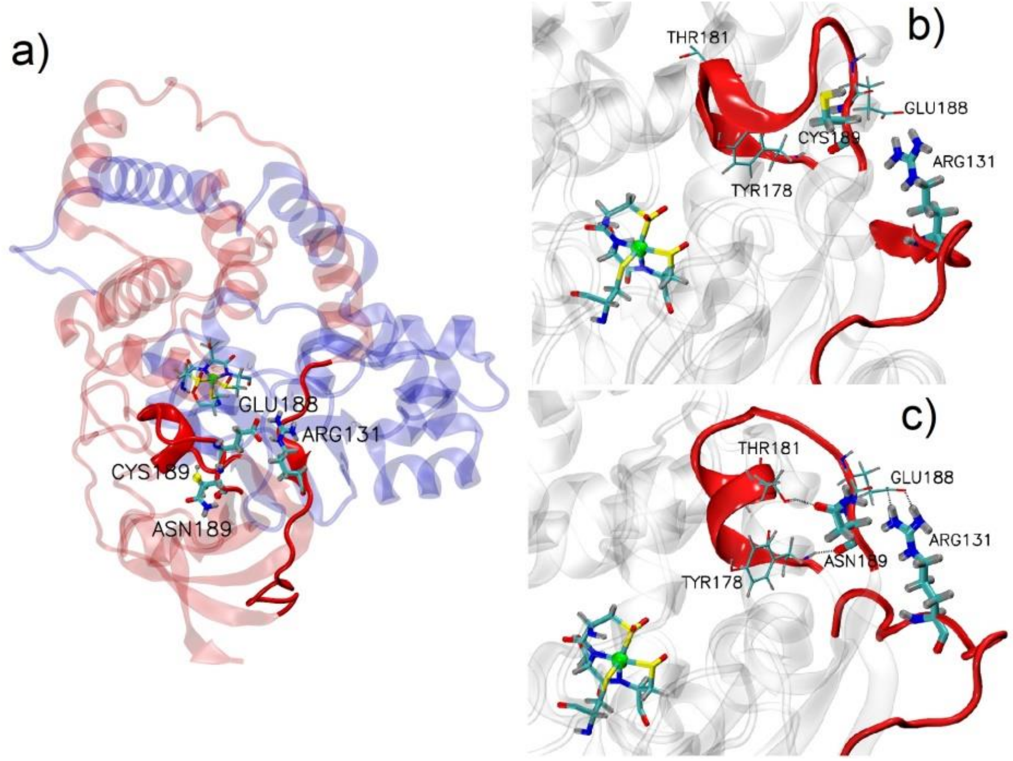

Figure S8. NHase with marked βCys189 in WT and βAsn189 in M10. Short α-helix (residues 178 to 183) and long loop (residues 127 to 142) are marked together with salt bridge βArg131-βGlu188 (a). The unfolding of the short helix in the case of WT is shown on panel (b). The hydrogen bond between βAsn189 and βThr181 present in simulations of M10 is shown in the panel (c).
